# Phylogenetic inferences reveal multiple intra- and interhost genetic diversity among bat rabies viruses circulating in northeastern Brazil

**DOI:** 10.1186/s42522-024-00124-6

**Published:** 2025-01-06

**Authors:** Larissa Leão F. de Sousa, Mariana Dias Guilardi, Junior Olimpio Martins, Bruna Stefanie S. Alves, Luiz Henrique S. Tibo, Patrícia da Silva-Antunes, Gustavo Cabral-Miranda, Débora Bellini Caldeira, Paulo Eduardo Brandão, Fabrício Souza Campos, Luiz Mário R. Janini, Ricardo Durães-Carvalho

**Affiliations:** 1 Department of Microbiology, Immunology and Parasitology, Federal University of São Paulo, São Paulo, SP Brazil; 2 Rabies Diagnosis Laboratory, Central Laboratory of Public Health - LACEN, Fortaleza, CE Brazil; 3 Interunit Bioinformatics Graduate Program, Institute of Chemistry, University of São Paulo, São Paulo, SP Brazil; 4 Department of Morphology and Genetics, Federal University of São Paulo, São Paulo, SP Brazil; 5 Institute of Biomedical Sciences, University of São Paulo (ICB/USP), São Paulo, SP Brazil; 6 Department of Medicine, Federal University of São Paulo, São Paulo, SP Brazil; 7 School of Veterinary Medicine, University of São Paulo, São Paulo, SP Brazil; 8 Virology Laboratory, Department of Microbiology, Immunology, and Parasitology, Institute of Basic Health Sciences, Federal University of Rio Grande do Sul, Porto Alegre, RS Brazil

**Keywords:** Bats, Rabies virus, Brazil, Intrahost genetic diversity, Interhost genetic diversity

## Abstract

**Background:**

Rabies, a lethal viral zoonotic disease, remains a significant global public health concern. In northeastern Brazil, in particular, its epidemiology is complex and dynamic, characterized by the presence of several reservoirs associated with human rabies infection.

**Methods:**

This study, conducted from June 2022 to July 2023, was part of a passive epidemiological surveillance initiative under Brazil’s National Rabies Surveillance Program. It investigated the presence of Rhabdovirus (RhabV) in 356 *postmortem* chiropteran brain samples using three diagnostic techniques for rabies and conducted an evolutionary study on both pan-RhabV- and pan-LYSSAV-positive PCR samples. The samples were collected from 20 bat species and different locations in the State of Ceará, an endemic region for the rabies virus (RABV). Rabies-positive samples were further explored through Bayesian, genetic distance mapping and recombination analyses.

**Results:**

From a total of 356 samples collected, 43 (12.07%) were positive for direct immunofluorescence (DIF) and 40 (11.23%) for mouse intracerebral inoculation (MIT) tests. Among the positive results, 40 samples were confirmed by both DIF and MIT, while 13 (3.65%) had inconclusive results for one or both techniques. Molecular assays identified 38 rabies-positive samples (10.67%). Members of the Molossidae and Phyllostomidae families had the highest prevalence, highlighting the role of insectivorous and frugivorous bats in the cycle and dynamics of rabies transmission. Phylogenetic reconstructions revealed three distinct and well-supported clusters and clades, indicating the cocirculation of different RABV lineages in the region and shedding light on both intra- and interhost diversity. We also demonstrated genetic distance among the RABV clusters and inferred that their common ancestor originated in Europe, later diversifying across continents. No recombination breakpoints were identified.

**Conclusions:**

This study highlights the dynamic nature of RABV evolution within individual bat hosts, contributing to the understanding of the genetic diversity of RABV variants found in several bat species in northeastern Brazil. This study provides crucial insights into viral transmission dynamics within and between different host species and is essential for designing effective rabies control and prevention strategies tailored to endemic regions.

## Background

Rabies is a zoonosis that leads to 100% lethality and can infect a wide variety of mammals [1]. In Brazil, over the past 13 years, 47 cases of human rabies have been reported, with 51.06% (24 cases) being transmitted by Chiropterans [2]. The rabies virus (RABV), a member of the genus Lyssavirus (LYSSAV), is distributed among several host species. In Brazil, particularly in the state of Ceará, an endemic region for rabies, domestic dogs and cats, as well as wild animals such as foxes, non-human primates (*Callithrix jacchus*) and bats, play a crucial role in the transmission and evolution of RABV [3, 4]. Such animals are pivotal to the ongoing spread and adaptation of the virus across Ceará and Brazil. Importantly, bats serve as the ancestral reservoir of rabies, with a wide-ranging diversity harboring well-established antigenic variants associated with *Desmodus rotundus*, *Tadarida brasiliensis* and *Lasiurus cinereus* [5].

RABV, belonging to the order *Mononegavirales*, family *Rhabdoviridae*, comprises 46 genera and 318 species identified in a wide range of hosts [6]. RABV is an enveloped virus with a bullet-shaped morphology and a negative-sense single-stranded RNA genome. Its genome varies in size from 10.8 to 16.1 kb and encodes a set of five proteins: nucleoprotein (N), phosphoprotein (P), matrix protein (M), surface glycoprotein (G) and RNA-dependent RNA polymerase (L) [7]. Errors in the RNA-dependent RNA polymerase may contribute to genetic heterogeneity in RABV populations, potentially enhancing the virus’s capacity to adapt to different hosts [8].

The presence of genetic heterogeneity has been demonstrated in RABVs, showing that mutations in populations of these viruses leads to adaptation within a new environment, resulting in new variants [9]. Some studies have identified a diversity of RABVs variants in bats distributed geographically, most likely related to the ecology of these animals [10, 11]. Nevertheless, both intra- and interhost diversity play pivotal roles in the persistence and evolution of RABV [12]. Conversely, interhost diversity pertains to how the virus behaves in different infected individuals and animals. This diversity contributes to the ability of viruses to evade host immune responses and adapt to different environments within the host [13].

Understanding rabies requires insight into the diversity among host species, as different reservoirs have unique transmission dynamics that pose varying public health risks [9]. Some studies have highlighted RABV’s notable ability to cross species barriers, leading to the development of distinct genetic lineages [8, 9, 14, 15]. However, there is a lack of studies reporting these aspects, including the differentiation of viral populations across different hosts. Ongoing research into the virus within its reservoir is crucial for understanding its behavior and the disease dynamics at a regional level, which is essential for informing public health strategies.

## Methods

### Sampling and viral screening

This study focused on rabies epidemiological surveillance through qualitative analysis of post-mortem animals as part of Brazil’s National Rabies Surveillance Program [16]. We sampled 356 brain tissues from 20 bat species across different regions in the State of Ceará, northeastern Brazil (Table 1). These samples were collected between June 2022 and July 2023. Bat species were identified based on morphological criteria [17]. To estimate species diversity and the abundance of taxa, we calculated the Shannon index [18] using the Omni Calculator platform (https://www.omnicalculator.com/ecology/shannon-index). Ethical approval was obtained from the Biodiversity Information and Authorization System (SISBIO) under no. 85149-1 and the UNIFESP Ethics Committee for Animal Experimentation under no. 3299080922.

**Table 1:** Diversity of bats circulating in Northeast Brazil included in the study, along with their respective diagnostic tests for rabies

Bat species	*n* (2022)	*n* (2023)	Location	Rabies diagnostic tests		
				DIF	MIT	pan-RhabV and -LYSSAV PCR assays
*Artibeus jamaicensis*	1		North	+	Inconclusive	+/+ (*n* = 1)
	1		West	-	-	+/+(*n* = 1)
*Artibeus lituratus*	3		North	-	-	-
	4	9	West	-	-	-
*Carollia perspicillata*	1		East	-	-	-
	1	1	North	-	-	-
	1	3	West	-	-	-
*Cormura brevirostris*		1	East	-	-	-
*Desmodus rotundus*		1	East	+	+	+/+ (*n* = 1)
		50	West	-	-	-
*Eumops glaucinus*	1		East	Inconclusive	Inconclusive	-
	1		East	-	-	-
	1		West	-	-	-
*Eumops perotis*		1	East	+	+	+/+(*n*= 1)
		11	East	-	-	-
		1	North	-	-	-
		15	West	-	-	-
*Glossophaga soricina*		1	East	-	-	-
	1	1	North	-	-	-
	1	10	West	-	-	-
*Molossidae* (*Unidentified species*)		1	East	-	-	-
*Molossus molossus*	45	37	East	-	-	-
	5	16	East	+	+	+/+ (*n* = 21)
	1	3	East	+	+	-
	1		East	+	Inconclusive	+/+(*n* = 1)
		1	East	Inconclusive	Inconclusive	+/+(*n* = 1)
		1	East	Inconclusive	-	-
		1	North	Inconclusive	-	-
	7	17	North	-	-	-
	1		North	-	-	+/+(*n* = 1)
	1		North	+	Inconclusive	-
		3	North	+	+	-
		3	North	+	+	+/+(*n* = 3)
		4	South	-	-	-
		1	South	Inconclusive	+	-
		1	South	+	+	-
	2	4	West	+	+	+/+(*n* = 6)
	1		West	-	-	+/+(*n* = 1)
	13	11	West	-	-	-
		3	West	Inconclusive	-	-
*Noctilio leporinus*		1	North	-	-	-
*Nyctinomops laticaudatus*	1		East	Inconclusive	-	-
*Peropteryx macrotis*		3	East	-	-	-
	1	2	West	-	-	-
*Phylloderma stenops*	1		East	-	-	-
		1	North	-	-	-
*Phyllostomus hastatus*	5	12	West	-	-	-
*Promops nasutus*	1		East	-	-	-
	1		East	Inconclusive	Inconclusive	-
*Pteronotus gymnonotus*		7	West	-	-	-
*Rhinophylla fischerae*		11	West	-	-	-
*Sturnira lilium*	2		West	-	-	-
*Uroderma bilobatum*		1	East	-	-	-
**Total**	106	250				38

*Abbreviations* DIF, direct immunofluorescence; MIT, mice intracerebral inoculation; RhabV, Rhabdovirus; LYSSAV, Lyssavirus

Samples were subjected to the WHO-recommended direct immunofluorescence (DIF) technique. Infection confirmation occurred through virus isolation via mouse intracerebral inoculation (MIT) [19]. These experiments were performed at the Rabies Diagnosis Sector in the Central Laboratory of Public Health (LACEN, Fortaleza-CE, Brazil). Subsequently, total RNA extraction was performed using the Zymo Quick-RNA™ Viral Kit, followed by cDNA synthesis with the High Capacity cDNA Reverse Transcription Kit (Applied Biosystems™). The reverse-transcribed cDNA was subjected to pan-RhabV and pan-LYSSAV PCR assays targeting the polymerase (260 bp) [20] and nucleoprotein (606 bp) genes [21], respectively. Amplicons were purified using the Zymo DNA Clean & Concentrator^®^-25 Kit, Sanger sequenced, and analyzed using BLASTn. Sequence ambiguities were resolved using the sangerseqR package (https://bioconductor.org/packages/release/bioc/html/sangerseqR.html) at a ratio of 1.0. The GenBank DNA sequences generated in the study (37 from the N gene and 35 from the L gene) have been deposited with accession numbers PP389288-PP389324 (N) and PP389325-PP389359 (L).

### Phylogeny

Evolutionary history was carried out using concatenated N (484 nt) and L (151 nt) gene sequences through the SeqKit platform [22], resulting in a dataset with 635 nt. This concatenation strategy, previously employed in our study [15], demonstrated increased robustness in phylogenetic statistics along the branches. Sequences were filtered using Sequence Cleaner, a Biopython-based software (https://biopython.org/wiki/Sequence_Cleaner).

We retrieved whole-genomes (*n* = 1.676) and nucleoprotein gene sequences (customized length) (*n* = 5.300) of RABV from GenBank, representing different geographical regions, collection years, and hosts. These datasets, which included our laboratory-produced sequences, comprised 5.337 sequences for the N gene and 1.711 for the L gene (Electronic Supplementary Material), totaling 7.048 sequences. Alignments were conducted using the MAFFT algorithm (https://www.ebi.ac.uk/Tools/msa/mafft/) and manually edited using Unipro UGENE v.49.1 [23] and AliView [24] softwares. The phylogenetic signal was investigated by Tree-Puzzle v.5.2 through the likelihood mapping analysis of 10.000 random quartets [25], and the substitution saturation index was assessed using DAMBE v.7.3.0 software [26], which indicated little saturation.

Global maximum likelihood (ML) phylogenies were reconstructed using FastTree v.2.1.7 software [27], employing the standard implementation GTR + CAT with 20 gamma distribution parameters and a mix of nearest-neighbor interchange (NNI) and subtree-prune-regraft (SPR) though 1.000 replicates of the Shimodaira-Hasegawa (SH-like) test. The history of rabies dispersal over time was inferred from a filtered dataset (*n* = 126) comprising distinct clusters identified in the global RABV N and L phylogenies (Electronic Supplementary Material).

Bayesian analysis was conducted in BEAST v.1.10 under an uncorrelated relaxed lognormal clock, assuming a SkyGrid model as the coalescent prior [28]. The “time at last transition point” was set to 50.0. Phylogeographic analysis was performed with discrete traits using a symmetric substitution model and the Bayesian stochastic search variable selection (BSSVS) algorithm [29]. A general time-reversible (GTR) +Γ4 Markovian nucleotide substitution model was chosen as the best-fit model. Two independent Markov chain Monte Carlo (MCMC) runs were performed for 500 million generations. Mixing and convergence (≥ 250 Effective Sample Size) were assessed using Tracer v.1.7 software after a 10% burn-in [30]. Spatiotemporal information was estimated using SpreaD3 [31], and the maximum clade credibility (MCC) tree was inferred using TreeAnnotator v.1.10. Node reliability was analyzed by posterior probability support values. Qualitative analysis of trees was performed with the DensiTree BEAST package (Electronic Supplementary Material). Phylogenetic trees were visualized using FigTree v.1.4.4 (http://tree.bio.ed.ac.uk/software/figtree/) and TreeViewer (https://treeviewer.org/) softwares.

#### Heatmap of RABV N and L gene multiclusters and recombination analysis

We utilized MEGA v.11 software [32] to calculate pairwise nucleotide genetic distances within the partial coding regions of the RABV N and L genes, focusing on the three distinct clusters identified in the reconstructed phylogenies (Electronic Supplementary Material). Next, we conducted a comparative analysis of the relative genetic divergence in intra- and interhost nucleotide variation. Heatmaps were generated using the Pheatmap package in R v.3.4 (https://cran.r-project.org/web/packages/pheatmap/index.html*).*

To investigate the presence of recombination breakpoints in the intra- and inter-clade phylogeny of rabies, the N and L genes were examined separately, using sequences retrieved from clusters and the respective RABV reference (GenBank accession NC_001542.1). A thorough exploratory recombination scan was carried out using the Recombination Detection Program (RDP) v.5 program to identify recombinants, as well as minor and major parental sequences within the RABV N and L dataset. Significance was determined by *p-*values ≤ 0.05. Only events deemed statistically significant across nine algorithms in the RDP were considered indicative of the in silico presence of recombination.

## Results

Our initial screening assessed a total of 356 bat brain samples for RABV antigens using direct immunofluorescence (DIF) technique, with 43 samples (12.07%) testing positive. All 43 positive samples were then subjected to the mouse inoculation test (MIT), which identified 40 (11.23%) as positive. Therefore, these 40 samples were positive by both DIF and MIT. Additionally, 13 samples (3.65%) yielded inconclusive results for either one or both of these techniques. The majority of our rabies-positive samples were from the Molossidae family, followed by species from the Phyllostomidae family. The prevalence of rabies was 10.6% (Table 2). Our Shannon index was 1.8, indicating a high level of taxon diversity in our collected samples. The data from Table 1 reveal that while one sample from *Molossus molossus* and another from *Artibeus jamaicensis* showed positive results in the DIF assay, their outcomes were inconclusive in the MIT. Notably, the sample from *M. molossus* yielded inconclusive results in both experiments. Furthermore, one sample from *A. jamaicensis* and two additional samples from *M. molossus* tested negative in both the DIF and MIT assays. Conversely, pan-RhabV-LYSSAV PCR assays detected 38 rabies-positive samples (10.67%), including those yielding inconclusive and negative results based on WHO-recommended techniques for rabies diagnosis.

**Table 2:** Number and percentage of bats sampled in 2022 and 2023 categorized by family, genus and species

Family, *Genus* and Species	Sample *n* (%)	Positive* *n* (%)	Rabies prevalence
**Emballonuridae**	7 (1.9)	0	-
*Cormura*			-
*Cormura brevirostris*	1	0	
*Peropteryx*			-
*Peropteryx macrotis*	6	0	
**Molossidae**	**216 (60.6)**	**35 (92.1)**	**16.2**
Unidentified species	1	0	
*Eumops*			3.22
*Eumops glaucinus*	3	0	
*Eumops perotis*	28	1	
*Molossus*			18.5
*Molossus molossus*	183	34	
*Nyctinomops*			-
*Nyctinomops laticaudatus*	1	0	
**Mormoopidae**	**7 (1.9)**	**0**	**-**
*Pteronotus*			-
*Pteronotus gymnonotus*	7	0	
**Noctilionidae**	**1 (0.2)**	**0**	**-**
*Noctilio*			-
*Noctilio leporinus*	1	0	
**Phyllostomidae**	**125 (35.1)**	**3 (7.8)**	**2.4**
*Artibeus*			11.1
*Artibeus jamaicensis*	2	2	
*Artibeus lituratus*	16	0	
*Carollia*			-
*Carollia perspicillata*	7	0	
*Desmodus*			1.9
*Desmodus rotundus*	51	1	
*Glossophaga*			-
*Glossophaga soricina*	14	0	
*Phylloderma*			-
*Phylloderma stenops*	2	0	
*Phyllostomus*			-
*Phyllostomus hastatus*	17	0	
*Promops*			-
*Promops nasutus*	2	0	
*Rhinophylla*			-
*Rhinophylla fischerae*	11	0	
*Sturnira*			-
*Sturnira lilium*	2	0	
*Uroderma*			-
*Uroderma bilobatum*	1	0	
**Total**	**356**	**38**	**10.6**

*Positive results are based on PCR analysis

**Fig. 1:**
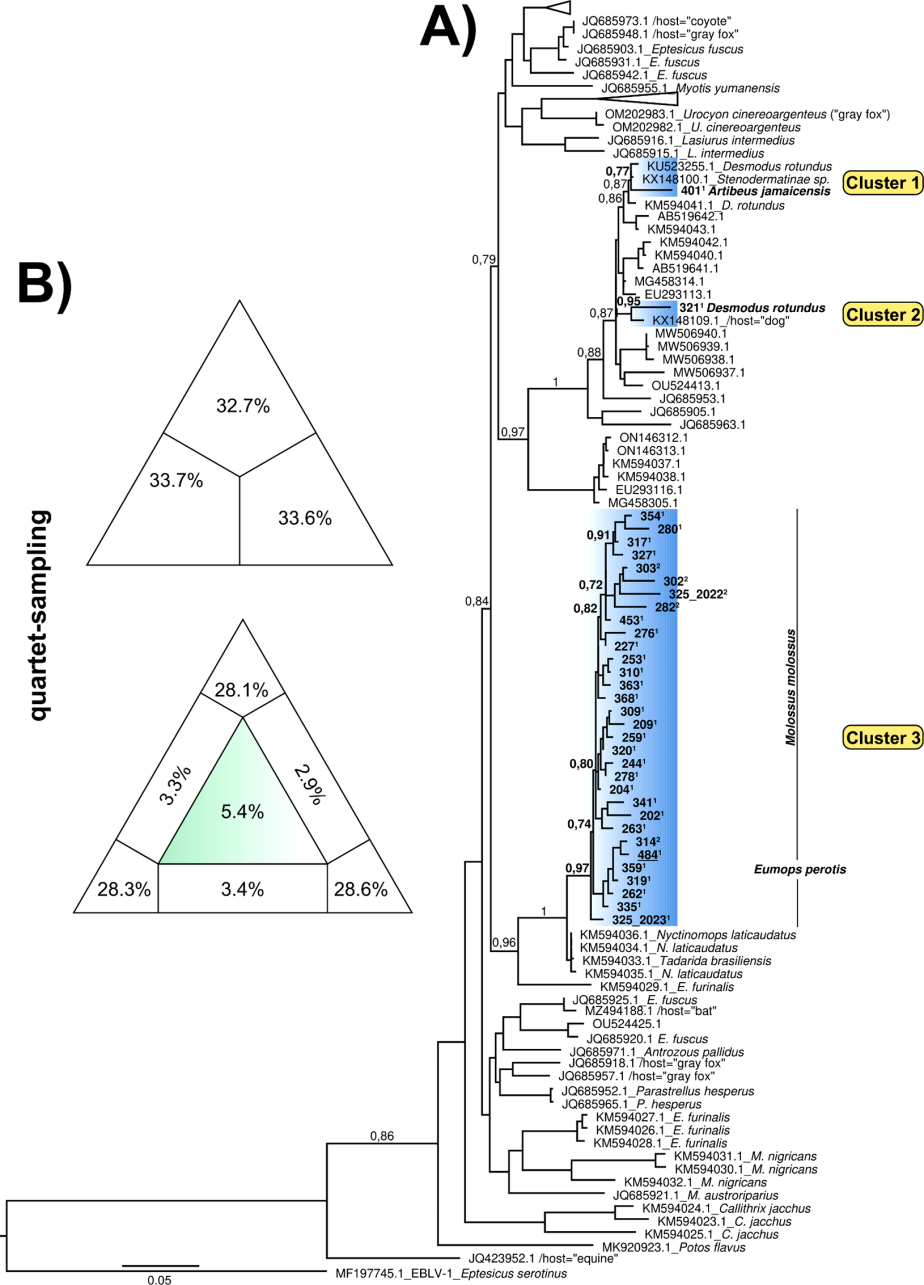
Concatenated maximum likelihood (ML) phylogenetic tree (**A**) and likelihood mapping (**B**) based on the rabies virus (RABV) N and L gene datasets. Multiple host-RABVs phylogenetic tree highlighting in blue the clusters found in this study, alongside those from different animal species (**A**), and the phylogenetic signal (**B**) of the concatenated RABV N and L gene dataset. Samples with superscript numbers in their IDs refer to sequences obtained from the original source (**ID**^1^) and biological proof in mouse (**ID**^2^), respectively. Support values based on SH-like test are indicated along the branches. The scale bar indicates the number of nucleotide substitutions per site (**A**). Within the triangles, dots represent the likelihood of possible unrooted topologies for each quartet, with numbers indicating phylogenetic noise (star-like trees). If more than 30% of the mapping falls into the center of the triangle, the data are considered unreliable for phylogenetic inference (**B**). Host species retrieved from GenBank are listed at the tips of the branches

Maximum likelihood-based analyses of the concatenated RABV N and L genes revealed three distinct and well-supported rabies multihost clades and subclades (Electronic Supplementary Material; Fig. 1). In the first subclade, designated Cluster 1, our sample (401) clustered with RABV variants detected in *D. rotundus* (GenBank accession no. KU523255.1) and *Stenodermatinae* sp. (GenBank accession no. KX148100.1), both from French Guiana, reported in 2010 and 2009, respectively. Conversely, the subsequent subclade, Cluster 2, which received statistical support of 0.95, included our sequence (321) grouped with a RABV variant from a canine host from Brazil dated to 1986 (GenBank accession no. KX148109.1).

The latter (Cluster 3), composed of a phylogenetically distinct and monophyletic clade, included a set of sequences from our laboratory, primarily RABV from *M. molossus.* These sequences were closely related to the RABV sequences found in *Nyctinomops laticaudatus* (GenBank accession no. KM594034-6) and *T. brasiliensis* (GenBank accession no. KM594033.1), both of which are Brazilian bat isolates from 2010. Additionally, this cluster included RABV from *Eptesicus furinalis* (GenBank accession no. KM594029.1), which originated in Brazil in 2006 and was identified as a common ancestor (Fig. 1).

**Fig. 2:**
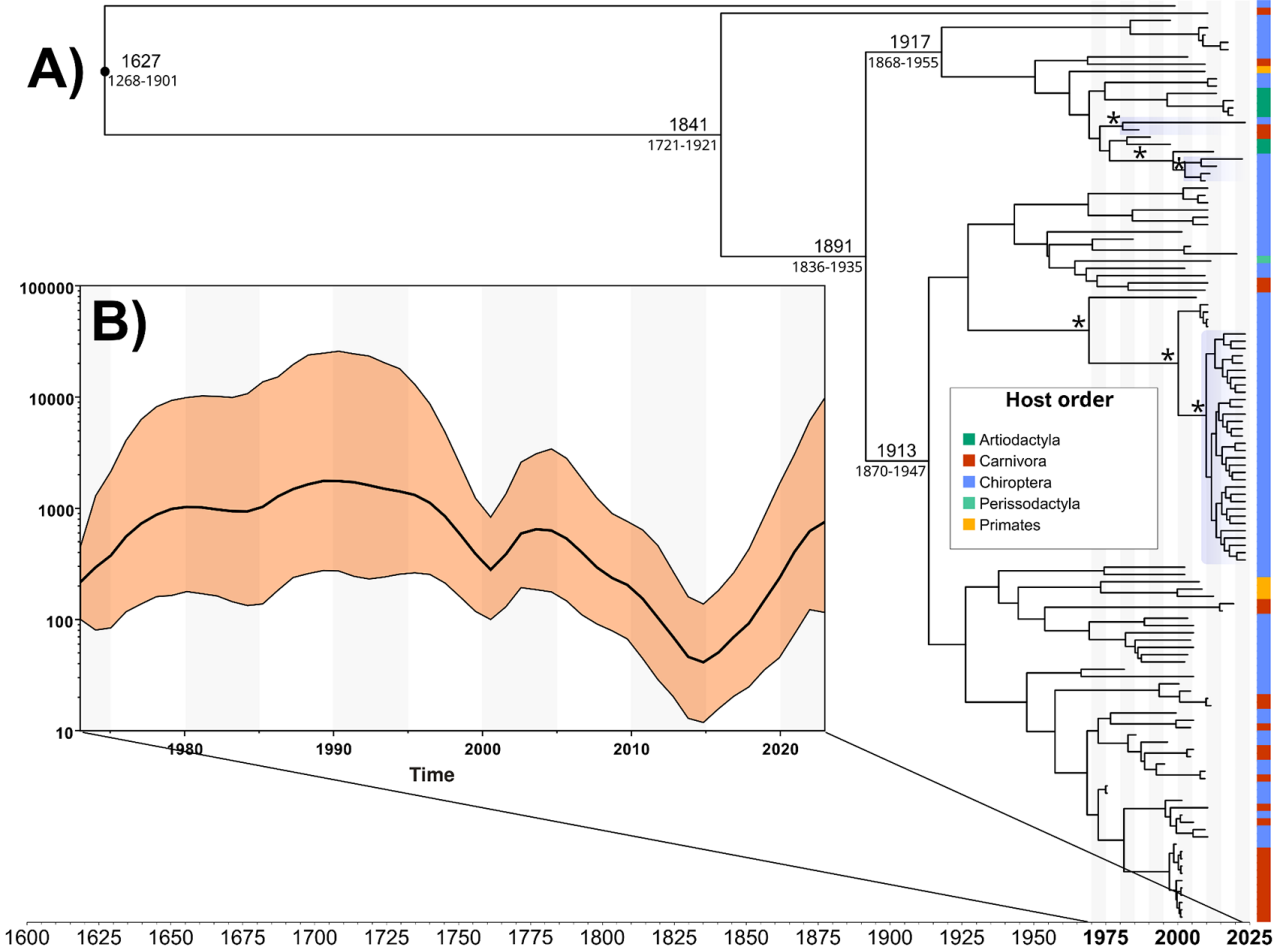
Maximum clade credibility (MCC) phylogenetic tree (**A**) and Skygrid reconstruction (**B**) inferred for rabies virus (RABV) based on concatenated N and L sequences. Phylogenetic tree illustrating the evolutionary relationships among RABVs sampled from distinct host species (**A**). Different host orders are color-coded for clarity, as indicated on the right vertical bar. Clusters of RABVs identified in the study are highlighted in light blue. The median age of each node, along with the 95% highest posterior density (HPD) interval, indicates the estimated timing of divergence events. Asterisks denote posterior probability values equal to or greater than 90%. The Skygrid reconstruction shows the population dynamics of RABVs over time. The y-axis represents the effective population size, with black lines indicating the means and the shaded orange area the 95% HPD interval (**B**)

**Fig. 3:**
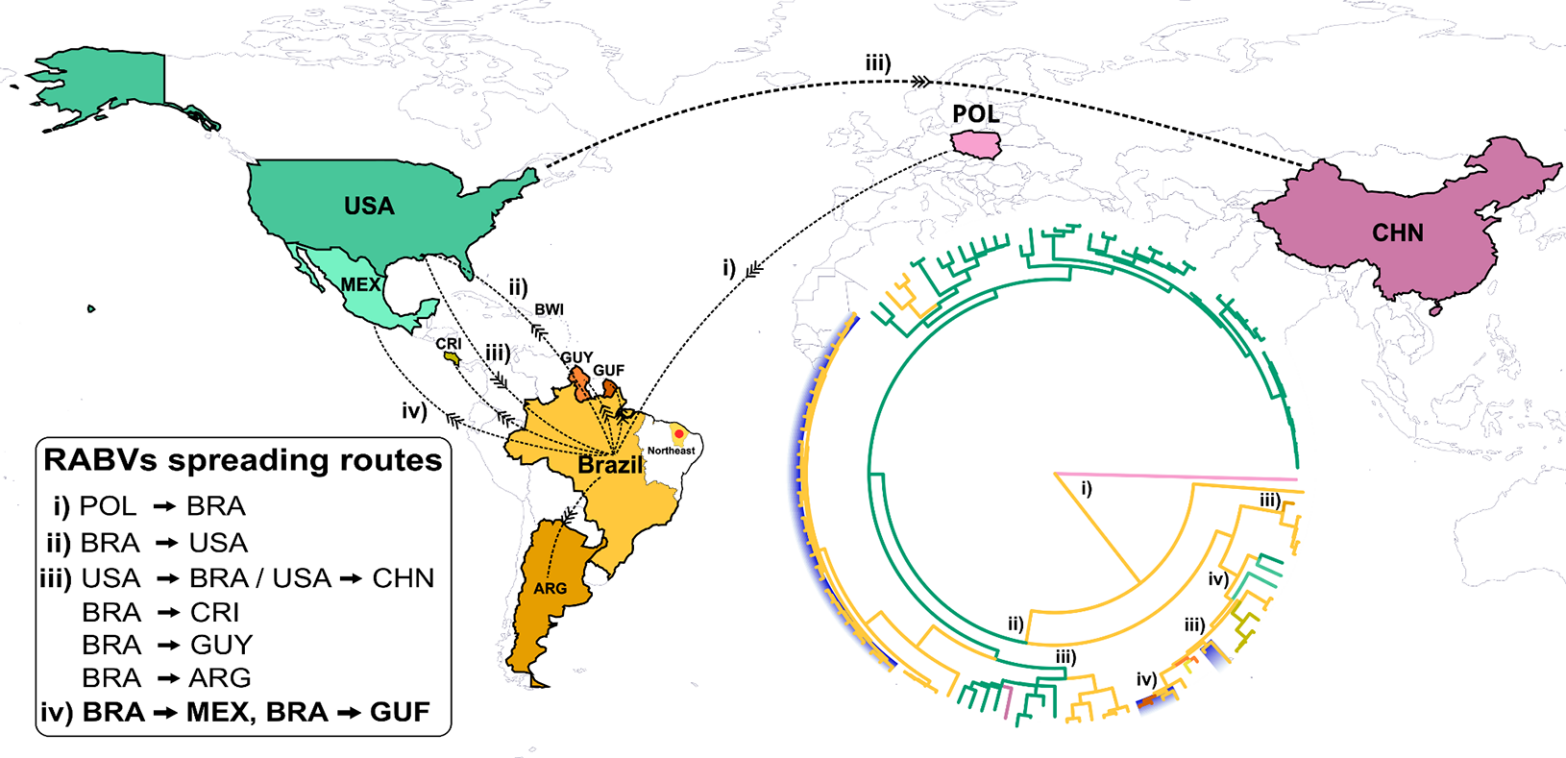
Map illustrating the temporal and geographic dispersal of the rabies virus (RABV) based on the maximum clade credibility tree (see Fig. 2). Major RABV dispersion events are indicated by Roman numerals (i)-(iv), representing distinct stages characterized by simultaneous transition routes. The branches of the circular phylogenetic tree are color-coded according to their respective countries. The state of Ceará, located in northeastern Brazil, place where the samples were collected, is highlighted by a red circle. The clusters of RABV identified in the study are highlighted in light blue in the phylogenetic tree on the right side. Country abbreviations: ARG, Argentina; BRA, Brazil; BWI, British West Indies; CHN, China; CRI, Costa Rica; GUF, French Guiana; GUY, Guyana; MEX, Mexico; POL, Poland; USA, United States of America

The Bayesian analysis reinforced the findings from the maximum likelihood (ML) multicluster inferences, achieving a posterior probability equal to or greater than 90% (Fig. 2). Moreover, a notable observation pertains to the temporal dynamics of both intra- and interhost viral diversity, as evidenced by the scaled phylogenetic branch length, indicating evolutionary changes over time for RABV among different host orders and regions. The reconstruction of population dynamics over a fifty-year span from 1973 to 2023 revealed distinct fluctuations, highlighting the dynamic nature of RABV spread and showing variations in population size and diversity over time (Fig. 2). Our findings further suggest the common ancestry of RABV originating from Europe, followed by subsequent diversification and spreading to different countries across the American continent (Fig. 3). The consistency of our findings across a broad range of bat species highlights the robustness and representativeness of our data. Such reproducibility has enabled us to delineate specific clusters of RABV lineages circulating among different bat species, providing a deeper understanding of the intra- and interhost dynamics and diversity of the virus.

**Fig. 4:**
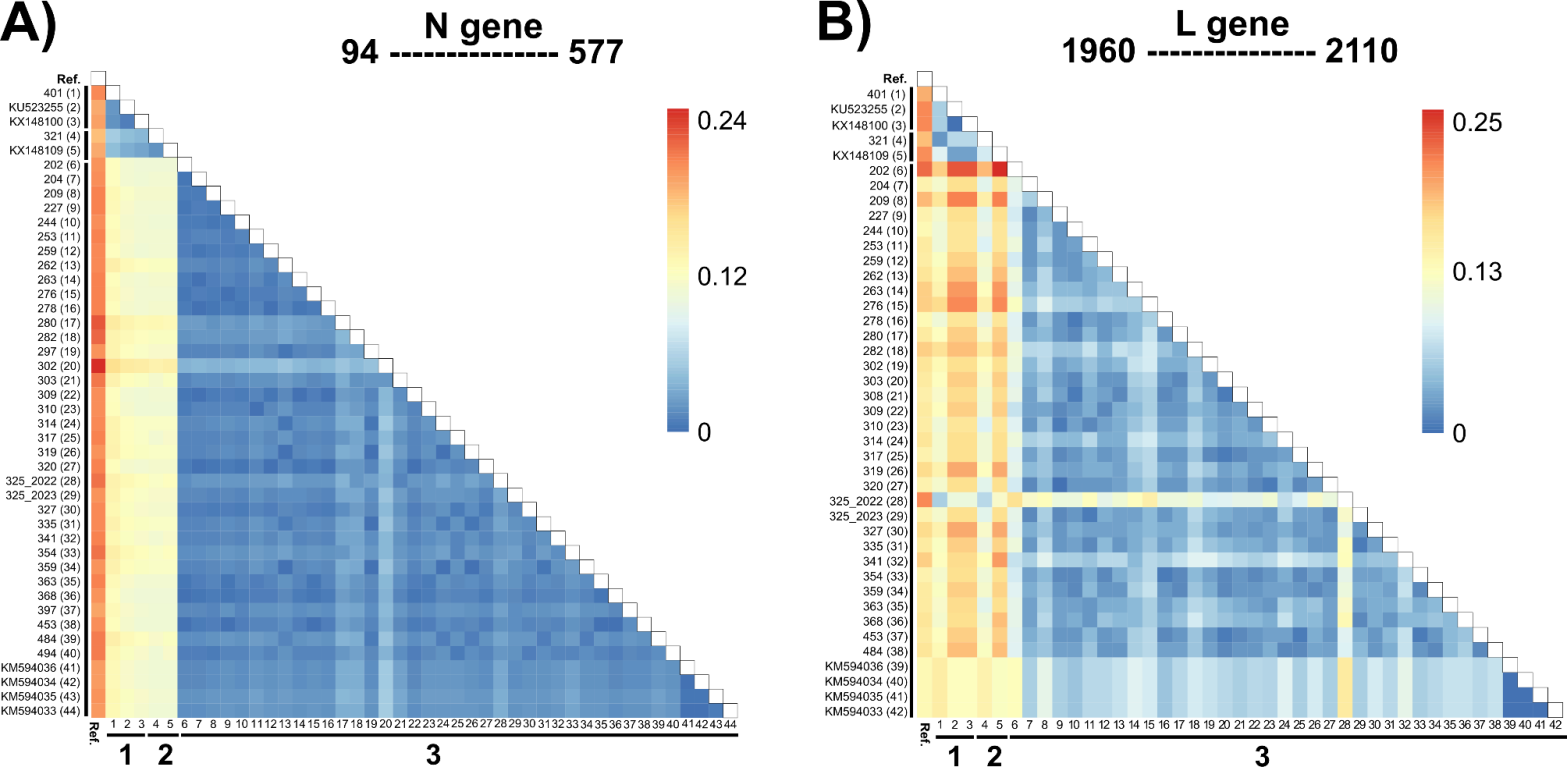
Pairwise genetic distance heatmaps for the partial coding regions of the rabies virus (RABV) N and L genes corresponding to the three clusters recovered from the phylogeny shown in Fig. 1. Pairwise genetic distances calculated for the RABV N (**A**) and L (**B**) gene sequences. The legend provides values in substitutions per site, with red indicating the highest values of paired genetic distance and blue indicating values close to zero. The RABV reference sequence (GenBank accession NC_001542.1) is denoted as “Ref.”

Lastly, analysis of genetic distance, focusing on both the nucleoprotein and polymerase genes, revealed a greater divergence between sequences in Clusters 1 and 2, as depicted in Fig. 4. Notably, the nucleoprotein gene displayed greater relative genetic distances between Clusters 1 and 2 compared to Cluster 3 (ranging from 0.11 to 0.17), while exhibiting minimal variation between Clusters 1 and 2 (Fig. 4A). Conversely, the polymerase gene sequences exhibited the most substantial genetic distance variations across all clusters (Fig. 4B). Furthermore, we found no in silico evidence of recombination events associated with our laboratory-generated sequences. Therefore, in accordance with our methodological criteria, these findings did not warrant further exploration.

## Discussion

Our study investigated the presence of Rhabdovirus (RhabV) and conducted an evolutionary analysis on 38 rabies-positive samples out of 356 deceased bat brain specimens obtained through molecular assays. Notably, our investigation revealed the highest positivity rate among samples from the Molossidae family, shedding light on the potentially overlooked role of insectivorous bats in the cycle and dynamics of rabies transmission [33, 34]. Traditionally, rabies detection has been focused on WHO-recommended techniques such as DIF and MIT, which are widely practiced in the field [19]. However, our results revealed that samples initially yielding negative or inconclusive outcomes in these assays tested positive in molecular experiments, a confirmation later validated through sequencing. This emphasizes the crucial role of molecular epidemiology, not only in corroborating results from DIT and MIT tests but also in identifying positive samples that might have escaped detection through these conventional methods [35].

Here, we also identified three well-defined and strongly supported RABV multihost clusters and clades, indicating the presence of distinct phylogenetic groups. This observation underscores the extensive genetic diversity among cocirculating RABV lineages in the State of Ceará, an endemic region for rabies, and provides insights into both intra- and interhost RABV diversity. For instance, a molecular epidemiological and phylogenetic study on rabies conducted in 2011, focusing on the nucleoprotein-encoding gene and involving nonhematophagous bats circulating in Brazil, identified distinct clusters of antigenic and genetic variants associated with hematophagous bats *D. rotundus* [36].

Globally, seven primary lineages of RABV have been identified, with various variants emerging within each lineage. These variants are associated with specific mammalian hosts and distinct geographical regions [37]. The mammalian orders Carnivora (carnivores) and Chiroptera (bats) are recognized as key reservoirs for the maintenance and transmission of rabies worldwide [38]. In Brazil, RABV is linked to five specific variants: AgV1 and AgV2, found in domestic dogs; AgV3, associated with hematophagous bats *D. rotundus*; and AgV4 and AgV6, identified in insectivorous bats *T. brasiliensis* and *L. cinereus*, respectively. Additionally, a variant phylogenetically related to AgV2 is associated with wild canids (*Cerdocyon thous*, bush dog), while the antigenic variant AgVCN is linked to non-human primates (*C. jacchus*, white-tufted marmoset) [39].

The emergence of novel RABV lineages is a dynamic process closely related to the host genus and/or species. The adaptability of particular RABV lineages across different hosts relies on factors such as close interspecies contact as well as their phylogenetic proximity [38, 39]. For instance, previous studies based on the RABV N gene in bats circulating in Brazil have demonstrated that distinct RABVs clades are associated with specific mammalian hosts [37, 40, 41]. Consistent with these findings, our concatenated phylogenetic analyses also revealed distinct clusters and subclades among RABV strains sampled from distinct hosts. Also, genetic distances analysis of the N gene in these studies has shown variability, ranging from less than 0.10 to 0.21 [37, 41]. However, by adopting a multicluster approach that captures fragment-oriented genetic variation in RABV across various bat species, our data closely align with these values. We observed genetic distances ranging from 0.05 to 0.17 among sequences from Cluster 1 and those from Clusters 2 and 3, which is lower than the corresponding values for the RABV L gene (Electronic Supplementary Material).

Further analysis conducted in Brazil identified two distinct clades based on the L gene associated with canid and bat hosts [41]. This observation also corresponds with the findings reported by De Souza et al. (2017) [40] regarding the N gene. Notably, the N protein of RABV exhibits remarkable conservation compared to the other five viral proteins, making it a commonly utilized target in phylogenetic and phylogeographic investigations [42]. However, by using a concatenation approach, our results offered additional insights by revealing multiple layers of intra- and interhost genetic diversity.

Bayesian analysis provided additional support for the maximum-likelihood multicluster inferences of RABV, indicating a shared ancestry originating from Europe, followed by subsequent diversification across countries from the American continent. Our result supports the findings of a study conducted by Kobayashi et al. (2011) [43]. Furthermore, another phylogenetic investigation of rabies revealed groupings that correlate with geographical origin relative to host species, suggesting that RABV may possess the ability to readily traverse boundaries between different species [44]. The ability of viruses to traverse species boundaries over evolutionary timescales spanning millions of years is a common characteristic among viruses, although the extent and frequency of such transmission can vary among different viral families [45, 46].

Understanding the epidemiology and molecular characteristics of viruses with zoonotic potential is imperative, particularly for RNA viruses, which are prone to emergence due to their adaptive traits stemming from genome plasticity [47]. The dual dynamics of intra- and interhost diversity within the RABVs underscore the importance of ongoing surveillance and research to monitor genetic changes, evaluate the risk of spillover events, and develop effective control strategies. Gaining insight into the mechanisms underlying the cross-species transmission and host adaptation of RABV remains a pivotal aspect of continuous efforts aimed at reducing and eradicating rabies.

Although our study provides valuable insights into the genetic diversity and evolutionary dynamics of RABVs circulation in bats from northeastern Brazil, some limitations should be acknowledged. First, our sample size, although comprehensive, does not fully represent the entire bat population in the region. Additionally, the focus on deceased bat specimens may not capture the full spectrum of RABV diversity, which tends to be greater. Moreover, while our phylogenetic analyses are consistent and shed light on the evolutionary relationships among RABVs, they can be limited by the availability of sequences deposited in public databases. These limitations highlight the need for further research to elucidate the complexities of intra- and interhost RABV dynamics in distinct bat populations.

## Conclusions

This study underscores the dynamic evolution of RABV within individual bat hosts and provides insights into its genetic diversity across various bat species from northeastern Brazil. Our findings also shed light on the intra- and interhost diversity and transmission dynamics of RABVs over time. Understanding these dynamics is crucial for developing targeted interventions, such as vaccination campaigns and wildlife management strategies, to mitigate the impact of this lethal zoonotic disease on both human and animal populations.

## Data Availability

All the data generated and analyzed in this study are included in the main text, tables, and figures. Additional data and details are available at https://github.com/rduraescarvalho/rabies (folder Rabies 2).

## Abbreviations

BEAST: Bayesian Evolutionary Analysis Sampling Trees
DIF: Direct Immunofluorescence
G: Surface glycoprotein
GTR: General Time Reversible
LACEN: Central Laboratory of Public Health
LYSSAV: Lyssavirus
L: RNA-dependent RNA polymerase
M: Matrix protein
MCC: Maximum clade credibility
MCMC: Markov Chain Monte Carlo
MIT: Mouse inoculation test
MEGA: Molecular Evolutionary Genetics Analysis
N: Nucleoprotein
NNI: Nearest-Neighbor Interchange
P: Phosphoprotein
PCR: Polymerase Chain Reaction
RABV: Rabies virus
RDP: Recombination Detection Program
RhabV: Rhabdovirus
SH-like: Shimodaira-Hasegawa
SPR: Sub-Tree-Prune-Regraft
WHO: World Health Organization
